# Comparison of Solid-Water Partitions of Radiocesium in River Waters in Fukushima and Chernobyl Areas

**DOI:** 10.1038/s41598-017-12391-7

**Published:** 2017-09-29

**Authors:** Yoshio Takahashi, Qiaohui Fan, Hiroki Suga, Kazuya Tanaka, Aya Sakaguchi, Yasuo Takeichi, Kanta Ono, Kazuhiko Mase, Kenji Kato, Vladimir V. Kanivets

**Affiliations:** 1 Department of Earth and Planetary Science, The University of Tokyo, Hongo 7-3-1, Bunkyo-ku, Tokyo, 113- 8654 Japan; 2 Institute of Materials Structure Science, High-Energy Accelerator Research Organization (KEK), Oho, Tsukuba, Ibaraki, 305-0801 Japan; 3 Northwest Institute of Eco-Environment & Resources, CAS, 382 West Donggang Road, Lanzhou, Gansu 730000 China; 4 Department of Earth and Planetary Systems Science, Hiroshima University, Kagamiyama, Higashi-Hiroshima, Hiroshima, 739-8526 Japan; 5 Advanced Science Research Center, Japan Atomic Energy Agency, Tokai, Ibaraki, 319-1195 Japan; 6 Center for Research in Isotopes and Environmental Dynamics, University of Tsukuba, 1-1-1 Tennodai, Tsukuba, Ibaraki, 305-8577 Japan; 7 Department of Geosciences, Faculty of Science, Shizuoka University, 836 Ohya, Suruga-ku, Shizuoka, 422-8529 Japan; 8 Ukranian Hydrometeorological Institute, Nauka, 37, Kiev, Ukraine

## Abstract

Adsorption of radiocesium (RCs) on particulate matters in aquatic environment is important to understand its mobility and bioavailability. We here focused on factors controlling partition of RCs on particulate matters and sediments in Kuchibuto (Fukushima) and Pripyat (Chernobyl) Rivers, though RCs level in water was much smaller than WHO guideline. Moreover, Cs speciation and organic matter-clay mineral interaction were studied: (i) extended X-ray absorption fine structure showed that the contribution of outer-sphere complex of Cs on particulate matters is larger in Chernobyl than in Fukushima and (ii) scanning transmission X-ray microscope revealed larger association of humic substances and clay minerals in Chernobyl partly due to high [Ca^2+^] in the Pripyat River. Consequently, RCs is more soluble in the Pripyat River due to weaker interaction of RCs with clay minerals caused by the inhibition effect of the adsorbed humic substances. In contrast, particulate matters and sediments in the Kuchibuto River display high adsorption affinity with lesser inhibition effect of adsorbed humic substances. This difference is possibly governed by the geology and soil type of provenances surrounding both catchments (Fukushima: weathered granite; Chernobyl: peat wetland and carbonate platform) which leads to high concentrations of organic matter and Ca^2+^ in the Pripyat River.

## Introduction

A large amount of radiocesium (RCs) dispersed by the Fukushima Daiichi Nuclear Power Plant (FDNPP) accident and deposited in Fukushima area has migrated through river water on land^1,2^. In aquatic environment such as in river water, the biggest concern is the accumulation of RCs into fish through the food web because of their importance in the human diet^3,4^. The beginning of the food web is the uptake of RCs by aquatic microorganisms such as planktons, and the amount of intake is controlled by the dissolved fraction of RCs. Thus, solid-water partition of RCs is of particular importance in aquatic environments^2,4^.

Various studies have shown that RCs in river waters in Fukushima area are highly insoluble in terms of solid-water partition: more than 70% of RCs (i.e., dissolved fraction <30%) are strongly adsorbed on particulate matters in river waters^1,5,6^ primarily due to the high affinity of Cs for phyllosilicate minerals consisting of vermiculite-like structures^7,8^. The particulate matters in this manuscript are the solid materials suspended in the river water which was collected by the filtration with 0.45 μm membrane filter. The adsorbed fraction further increases temporally in river waters possibly because of aging effect, wherein RCs become slowly and irreversibly fixed into phyllosilicates in soil particles, which carry RCs into river systems^4,6^.

By contrast, the opposite case was observed in Pripyat River in Dnieper River reservoir system near Chernobyl in relation to the Chernobyl Nuclear Power Plant (CNPP) accident in 1986. At least within two years after the accident, more than 80% of RCs existed in dissolved form in Pripyat River^9^. Although the dissolved fraction gradually deceased due to aging effect^4^, the dissolved fraction is more than 50% even after 1989. The contrasting behavior of RCs between Chernobyl and Fukushima is important in interpreting the subsequent migration of RCs and their incorporation into the food web. Thus, this study investigates the origin of different solid-water partitions of RCs in Pripyat and Kuchibuto (a tributary of Abukuma River) Rivers in Chernobyl and Fukushima areas, respectively.

A plausible explanation for the difference should be the mineral composition of the particulate matters, which affect the adsorption-desorption behaviors of Cs on particulate matters^4,10^, through strong interaction via formation of inner-sphere (IS) complex between Cs and siloxane in phyllosilicate minerals in particulate matters^7,11,12^. In particular, frayed edge site (FES) has been suggested to fix trace levels of Cs into phyllosilicate minerals^13–18^. Given that FES sites with high selectivity for Cs are abundant in phyllosilicate minerals, one possible reason for the contrasting trend in partition of RCs is the difference in mineralogy of particulate matters between Fukushima and Chernobyl watersheds.

On the other hand, RCs in organic soil area is more mobile in Pripyat catchment area^19–21^, suggesting that natural organic matter (NOM) lowers the affinity of RCs for soil particles. This fact is consistent with the findings obtained by extended X-ray absorption fine structure (EXAFS)^12,22^, which demonstrated that the presence of NOM largely inhibits Cs adsorption even onto illite and vermiculite with high abundances of FES and interlayer sites (=strong adsorption sites). NOM blocks access of Cs to these sites. As a result, Cs is adsorbed only on weak sites such as planer sites in phyllosilicates^12^ and presumably to the sites in NOM through formation of outer-sphere (OS) complex.

Hence, the effect of NOM has possibly induced the contrasting results on dissolved fraction of RCs between Chernobyl and Fukushima. Actually, peat soil is the main type of the soil surrounding Chernobyl area^20^, whereas weathered granite is dominant in Fukushima area which exhibits large capacity of FES for fixing RCs^7,8,23^. Thus, high NOM content is possibly another important factor responsible for the high dissolved fraction of RCs in Pripyat River. High mobility of Cs in organic catchment in Chernobyl area has been suggested^20,21,24^, but a more direct evidence is needed to demonstrate the effect of NOM. In this study, the contrasting RCs partition in Fukushima and Chernobyl watersheds is studied using various methods, such as EXAFS and scanning transmission X-ray microscope (STXM), which are employed to analyze particulate matters collected using the same protocol used for the samples in Fukushima (Kuchibuto River) and Chernobyl (Pripyat River).

## Results

### Characterization of particulate matters and river sediments

**Figure 1:**
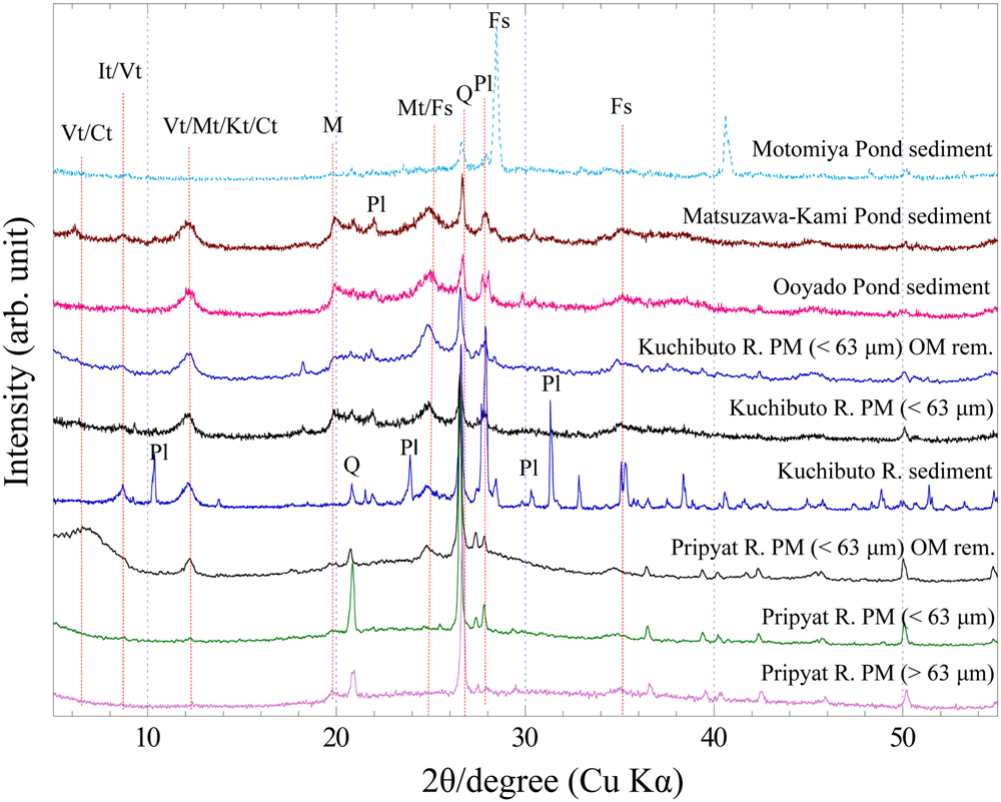
XRD patterns of particulate mattes (PM) and sediments from Chernobyl (Pripyat River) and Fukushima (Kuchibuto River, Ooyado Pond, Matsuzawa-Kami Pond, and Motomiya Pond). Q: Quartz; Vt: vermiculite; It: illite; Mt: montmorillonite; Ct: chlorite; Kt: kaolinite; M: mica; Pl: plagioclase; Fs: feldspar.

Figure 1 shows XRD patterns of particulate matters and sediments obtained from Chernobyl and Fukushima watersheds. Two peaks at 27° and 28° observed for all sediments and particulate matters are attributed to quartz and plagioclase, respectively^25^. Small peaks of mica and feldspar were observed at 19.7° and 35°, respectively. Compared with that in particulate matters obtained from Pripyat River, higher contents of micaceous minerals were found at 8.5° and 12.2° in sediment and particulate matters obtained from Kuchibuto River (Fukushima): these minerals include vermiculite, montmorillonite, and chlorite. Sediment obtained from Kuchibuto River contain larger amounts of plagioclase compared with particulate matters obtained from Kuchibuto River, suggesting that clay minerals, given their small particle size, are the main minerals existing in particulate matters (Fig. 1).

To understand the effect of NOM on the solid-water partitions of RCs, sediments in two irrigation ponds in Fukushima were also studied here (Ooyado and Motomiya Ponds)^26^: the Ooyado Pond sediment sample has similar mineralogy to the sediment and particulate matters of Kuchibuto River, whereas main components of Motomiya Pond sediment are feldspar and quartz. Major ions and ^137^Cs concentrations and other water quality data for the rivers and ponds were summarized in Tables 1 and 2. As a viewpoint of radiation protection, activities of ^137^Cs in water samples are much lower than that in WHO guideline for drinking water quality (=10 Bq/kg; WHO, 2011)^27^.

**Table 1:** Sampling sites and dates with compositions of major cations, anions, total dissolved solids (TDS), ^133^Cs, ^137^Cs and DOC in river and pond waters.

Sampling site	Sampling date	pH	Na^+^ (mg/L)	NH_4_^+^ (mg/L)	K^+^ (mg/L)	Mg^2+^ (mg/L)	Ca^2+^ (mg/L)	Cl^−^ (mg/L)	NO_3_^−^ (mg/L)	SO_4_^2−^ (mg/L)	HCO_3_^−b^ (mg/L)	TDS (mg/L)	^133^Cs (ng/L)	^137^Cs (mBq/L)	*DOC* (mg/L)
Pripyat River (Chernobyl) (N: 51° 27′25″, E: 30° 00′36″)	26-Aug-2013	8.03	9.9	0.041	2.1	7.1	64	18	1.1	13	43	158	53	24.9 ± 1.6	19
Motomiya Pond (Fukushima) (N: 37° 31′54″, E: 140° 36′49″)	30-May-2013	8.86	17	D.L.^a^	3.2	3.0	16	22	0.8	32	28	122	14	63.6 ± 0.1	5.8
Ooyado Pond (Fukushima) (N: 37° 31′58″, E: 140° 29′30″)	31-May-2013	6.84	12	D.L.	0.6	3.6	26	17	D.L.	8.5	54	121	10	37.0 ± 0.8	4.1
Kuchibuto River (Fukushima) (N: 37° 34′51″, E: 140° 32′31″)	19-Aug-2012	7.59	8.0	D.L.	1.7	2.2	14	7.0	5.8	8.8	42	89.5	32	3.92 ± 0.01	1.2

^a^D.L.: Lower than detection limit; ^b^HCO_3_^−^ concentration was calculated from total inorganic carbon and pH.

Uncertainties of concentrations of Na^+^, NH_4_^+^, K^+^, Mg^2+^, Ca^2+^,Cl^−^, NO_3_^−^, SO_4_^2−^, and ^133^Cs are better than 3%.

Uncertainties of HCO_3_^−^ and *DOC* concentrations are better than 5%.

**Table 2:** Physicochemical and mineralogical properties of particulate matter and sediment samples.

Sampling site	Sample type	Particle size or depth	^137^Cs (solid) (Bq/g)	NOMs (wt.%)	Illite	Vermiculite	Mica	*K*_d_^a^ *(L/g)*	N*-*NH_4_^+^ (μmol/g)
Pripyat River (Chernobyl)	Particulate matters (PSP-1)	0.45–63 µm	1.72 ± 0.01	16.7	×^b^	×	○^c^	69.2	53.3
	Particulate matters (PSP-2)	> 63 µm	1.60 ± 0.01	14.3	×	×	○	64.3	N.A.^d^
Motomiya Pond (Fukushima)	Very surface layer of soft sediment	0–2 cm	9.05 ± 0.01	10.6	×	×	×	142	49.2
Ooyado Pond (Fukushima)	Very surface layer of soft sediment	0–2 cm	9.30 ± 0.01	5.9	○	○	○	251	39.3
Kuchibuto River (Fukushima)	Surface sediment	0–5 cm(63–125 µm)	0.8 ± 0.01	1.1	○	○	×	204	D.L.
	Particulate matters	> 0.45 µm	12.72 ± 0.01	10.6	○	○	○	3240	N.A.^e^

^a^Natural distribution coefficient of  ^137^Cs; ^b^×: Not contained; ^c^○: Contained; ^d^N.A.: Not analyzed; ^e^D.L.: Lower than determination limit.

Uncertainties of NOM content is better than 3%. Uncertainties of NH_4_^+^ content is better than 5%.

Organic carbon contents of particulate matters from Pripyat River were 16.7 Cwt.% and 14.3 Cwt.% for two particulate matter samples with different particle sizes (PSP-1 (0.45–63 µm) and PSP-2 (>63 µm) in Table 2), respectively, and this result was similar to that obtained from Motomiya Pond sediment (Table 2). Such high NOM content can lead to weak adsorption affinity for Cs as confirmed by the small partition coefficients (*K*_d_) calculated from ^137^Cs concentrations in solid phase (particulate matters or sediments) relative to those in aqueous phase in river or pond systems (Table 2). Generally, such high organic carbon content makes detection of mineral peaks in XRD difficult. Thus, XRD patterns after removal of NOM through H_2_O_2_ treatment (Fig. 1) were measured, and two peaks were observed at approximately 8.5° and 12.2°, suggesting presence of phyllosilicate minerals also in particulate matters of Pripyat River.

The Fukushima catchment is granite-rich^7,8,28^, whereas organic peat soils cover approximately 47% of the Pripyat catchment (total catchment area is 200 km^2^) in Pripyat marshes in Ukraine-Belarus border^20,21^. High NOM content possibly with lower content of clay minerals indicated that *K*_d_ and capacity of Cs is relatively small in particulate matters of Pripyat River (and in Motomiya Pond sediment).

### Possible effects of mineralogy and NOM on radiocesium interception potential (*RIP*), or Cs adsorption

**Figure 2:**
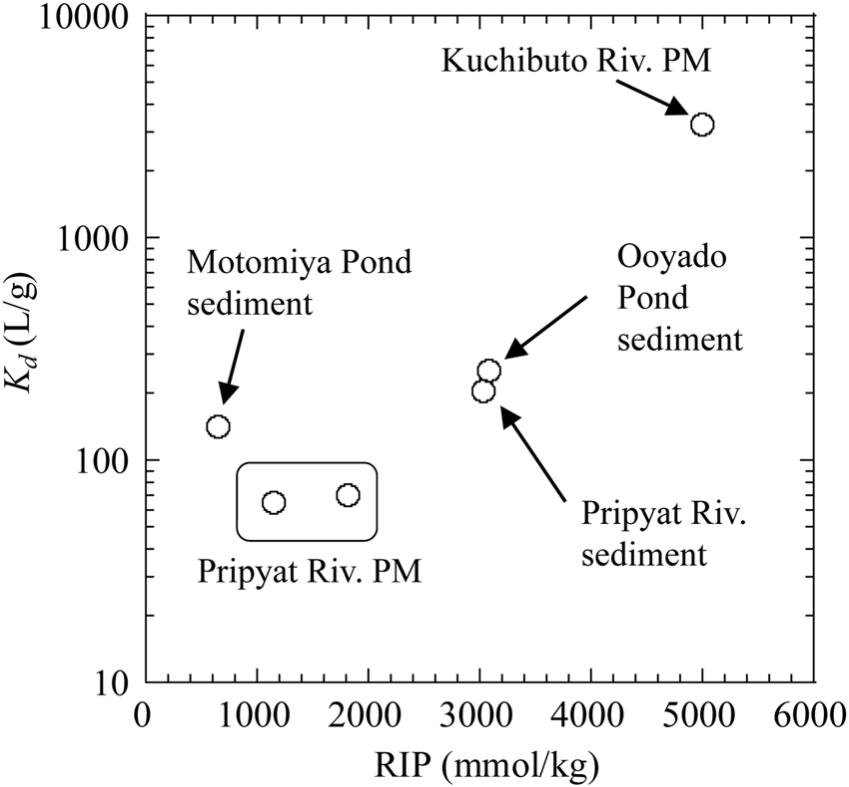
Relationship between *RIP* and *K*_*d*_ for particulate matters (PM) and sediments.

Figure 2 shows the radiocesium interception potential (*RIP*) and *K*_d_ of RCs sediments and particulate matters^28^ obtained from Fukushima and Chernobyl catchments. The *RIP* of particulate matters from Pripyat River is considerably smaller than those of particulate matters and sediments from Kuchibuto River and Ooyado Pond. *RIP* is considered as the most important factor for the retention of RCs in terrestrial and river systems^15–17,28,29^. Clay minerals normally display large *RIP* (above 6000 mmol/kg), except montmorillonite (~1000 mmol/kg)^29^. Variation in *K*_d_ is correlated with the variation in *RIP* (Fig. 2). Adsorbed fraction of ^137^Cs onto sediments and particulate matters is considerably larger in Kuchibuto River than in Pripyat River. Based on the definition of *RIP*, FES capacity is directly related to the difference in mineralogy of sediments and particulate matters obtained from Chernobyl and Fukushima, which includes effect of NOM content to inhibit formation of inner-sphere (IS) complex of Cs into the interlayer of the minerals^12,16,25^. The higher organic carbon content of particulate matters in Pripyat River (16.7 wt.% for PSP-1) than in Kuchibuto River (=10.6 wt.%) can explain the smaller RIP and *K*_*d*_ values for particulate matters in Pripyat River. The difference in organic carbon content is possibly caused by the larger dissolved organic content (*DOC*; Table 1) in Pripyat River (=19.0 mg/L) than in Kuchibuto River (=1.2 mg/L), reflecting their provenances. Motomiya Pond sediment also exhibits lower RIP and *K*_d_ possibly due to the higher abundance of NOM. These results suggest that high NOM content in the particulate matters or sediment is important in reducing Cs adsorption, which will be discussed below based on EXAFS results.

### X-ray spectroscopic study on Cs speciation: EXAFS

**Figure 3:**
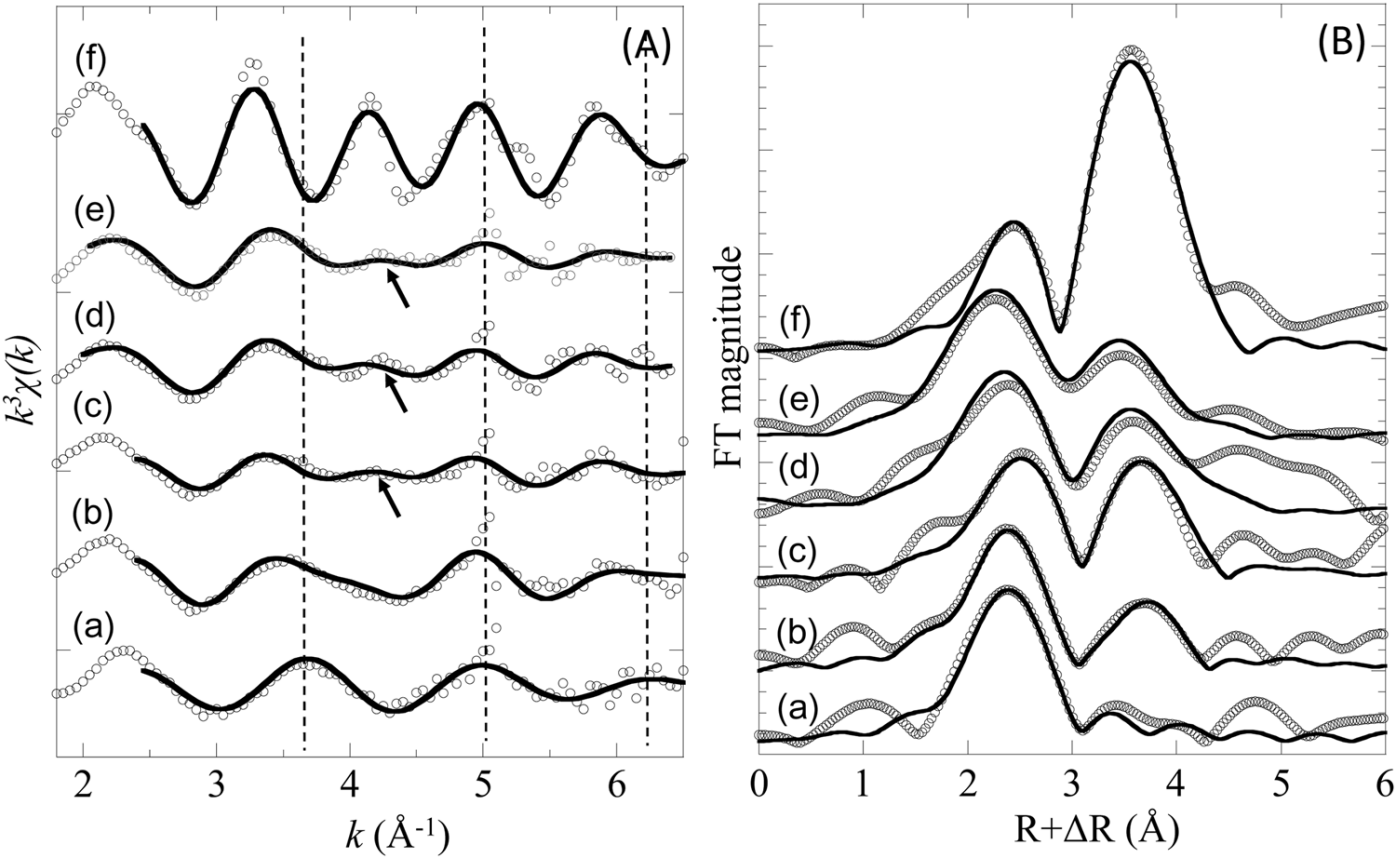
Cesium L3-edge EXAFS spectra in (**A**) k space and (**B**) R space. (a) hydrated Cs^+^ in water; (f) Cs adsorbed on vermiculite; Cs adsorbed on particulate matter in Pripyat River (b) before and (c) after the removal of organic matter; Cs adsorbed on particulate matter in Kuchibuto River (d) before and (e) after the removal of organic matter.

Adsorption species of Cs normally controls the mobility of Cs in the environment depending on the mineralogy and NOMs in particles^7,12,29^. In the present study, the adsorbed Cs species in sediments and particulate matters were estimated through EXAFS from the speciation of Cs added into the sample. The added concentration is much higher than natural level, but if IS complex is observed in this EXAFS analysis, the result will at least show that the particulate matters or sediment is capable of fixing Cs at lower Cs level such as for ^137^Cs in environment, since Cs should be adsorbed at more stable site when its concentration is lower. Figure 3A,B respectively show the Cs L3-edge EXAFS in k pace (*k* ^3^ *χ(k)* function) and the corresponding radial structural functions (RSFs; phase shift uncorrected) in R space for Cs adsorbed onto particulate matters and sediments. EXAFS spectra of CsNO_3_ solution (hydrated Cs^+^ ion) and Cs adsorbed on vermiculite are considered as two end members representing OS and IS complexes, respectively. Previous studies have confirmed that IS complex of Cs adsorbed onto vermiculite^12,30^.

The *k* ^3^ *χ(k)* function of Cs adsorbed onto particulate matters in Kuchibuto River (spectrum (d) in Fig. 3A; before removal of NOM) is obviously different from that of hydrated Cs^+^ ion (Fig. 3A), and this finding was also observed in Cs adsorbed onto sediments in Kuchibuto River. For example, the peak at approximately 3.7 Å^−1^ for the hydrated Cs^+^ ion shifted to approximately 3.3 Å^−1^ for the particulate matters in Kuchibuto River, and this phenomenon is due to the increase in peak intensity for IS Cs^+^ complex as seen in Cs adsorbed onto vermiculate, suggesting that contribution of IS complex is evident in Cs adsorbed onto particulate matters in Kuchibuto River. Similar results were found in RSF in Fig. 3B: (i) only one coordinated shell at (*R* + ∆*R*) ~2.4 Å was found in hydrated Cs^+^ ion, which originated from the hydration sphere of Cs^+^ and assigned to the OS complex; (ii) an intense shell at (*R* + ∆*R*) ~3.5 Å was determined in the spectrum (f) in Fig. 3B ^12,30,31^, suggesting that the IS complex is responsible for the high stability of Cs on minerals, such as vermiculite and illite. In practice, the two characteristic shells of the IS and OS complexes are successfully applied to distinguish Cs adsorption species in soils and sediments^7,12,29^. Expectedly, two shells in RSFs were observed for Cs adsorbed on particulate matters in Kuchibuto River (in this study) and Kuchibuto River sediments as reported by Fan *et al*.^25^ at *R* + ∆*R* ranges of 2.2–2.8 Å and 3.6–3.8 Å, respectively.

EXAFS for Cs adsorbed on particulate matters in Pripyat River showed contrasting results (spectrum (b) in Fig. 3). Absence of a small peak around k = 4.2 Å^−1^ in k space and lower contribution of the second shell in R space resulted in lower contribution of IS complex in the sample (Fig. 3). Relative ratio of the two shells (=CN_IS_/CN_OS_) is indicated in the coordination number (CN) of Cs-O shells in the second and first shells^12,29^. The CN_IS_/CN_OS_ ratio for particulate matters in Kuchibuto River was 0.78, considerably larger than 0.42 and 0.13 for particulate matters (PSP-1 and PSP-2, respectively) in Pripyat River (Table 3), demonstrating that IS complex of Cs is responsible for the higher *K*_*d*_ and RIP values of Cs in Kuchibuto River than in Pripyat River.

**Table 3:** Local structure of Cs adsorbed on river sediments and vermiculite using Cs L_III_-edge EXAFS^a^.

Sample	First shell^b^ (Cs-O_OS_)		Second shell^c^ (Cs-O_IS_/Si)			*ΔE*_0_	*R*_f_ (%)	*CN*_IS_/*CN*_OS_^b,c^
	*R* (Å)	*CN*	Shell	*R* (Å)	*CN*			
CsNO_3_ solution (hydrated Cs^+^)	2.98	8.0				−2.8	0.6	0
Particulate matters in Pripyat River (PSP-1)	2.98	6.7	Cs-O	4.13	2.8	−5.1	1.1	0.42
		Si-O	4.53	5.1				
Particulate matters in Pripyat River (PSP-2)	3.01	3.8	Cs-O	4.10	0.5	−4.4	5.3	0.13
		Si-O	4.54	4.5				
PSP-1 after removal of organic matters	3.06	3.9	Cs-O	4.14	4.8	−3.3	4.4	1.2
			Si-O	4.73	1.7			
Particulate matters in Kuchibuto River (KRSP)	3.03	4.9	Cs-O	4.09	3.5	−5.1	3.3	0.71
			Si-O	4.55	7.1			
KRSP after removal of organic matters	2.94	4.7	Cs-O	4.03	3.9	−8.4	0.4	0.83
			Si-O	4.44	7.6			
Vermiculite	3.08	1.3	Cs-O	4.13	5.8	−4.6	1.1	4.7
		Cs-Si	4.62	3.0				

^a^CN, coordination number; R, interatomic distance; ΔE0, threshold E_0_ shift; R_f_, residual factor. Errors in fit parameters were estimated to be generally ±0.02 Å for R, ±20% for CN as reported in O’Day *et al*.^50^.

^b^CN of first shell Cs-O was assigned to CN of the OS complex (CN_OS_).

^c^CN of second shell Cs-O was assigned to CN of the IS complex(CN_IS_).

^d^The two standards of CsNO_3_ and vermiculite were assumed to the end members of OS and IS complexes, respectively.

### X-ray spectroscopic study of NOM in particulate matters and sediments: STXM

**Figure 4:**
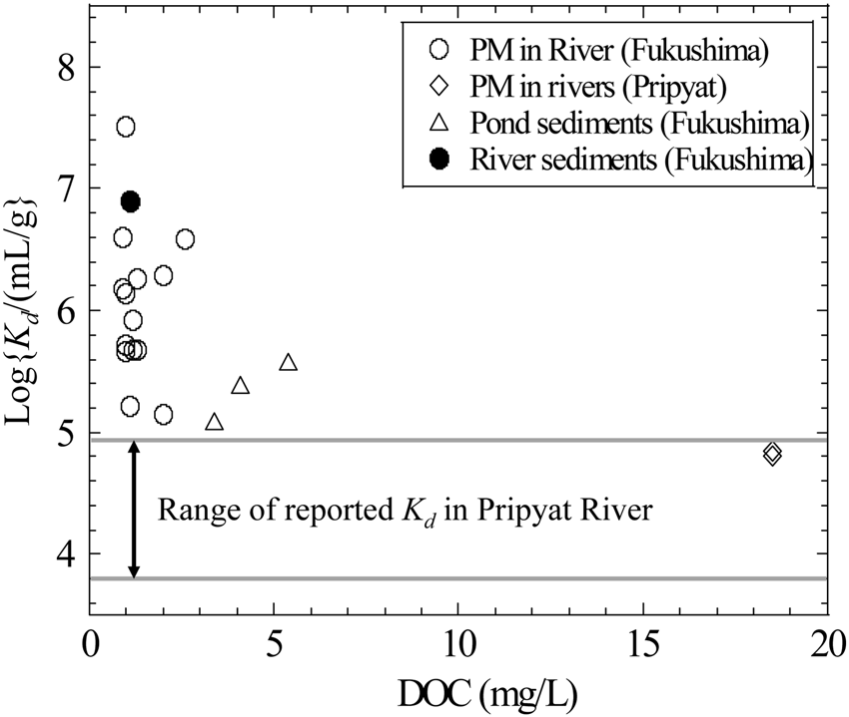
Natural partition coefficients of ^137^Cs as a function of dissolved organic carbon content (*DOC*) in Fukushima and Pripyat rivers and ponds.

Normally, chemical and mineralogical characteristics of particulate matters are similar to those of surface soil layer surrounding the rivers^5,32^. The Fukushima area is geologically classified as weathered granite and the Chernobyl as peat wetland^1,20,33^. Correspondingly, higher content of NOM content or *DOC* was observed in Pripyat River than in Fukushima watershed (Tables 1 and 2)^20,21^. Figure 4 shows the relationship between the natural *K*_d_ of ^137^Cs and *DOC* content in rivers and ponds (two ponds in Table 1 with data of Matsuzawa-kami Pond in Yoshimura *et al*.^26^) in Fukushima and Chernobyl areas, as well as the results reported in Sakaguchi *et al*.^5^ *K*_d_ of ^137^Cs adsorbed on particulate matters sharply decreases with increased *DOC* possibly due to the inhibition effect of NOM on the access of Cs to strong adsorption sites, such as FES and interlayer site in phyllosilicate minerals^12,14,20,34^.

**Figure 5:**
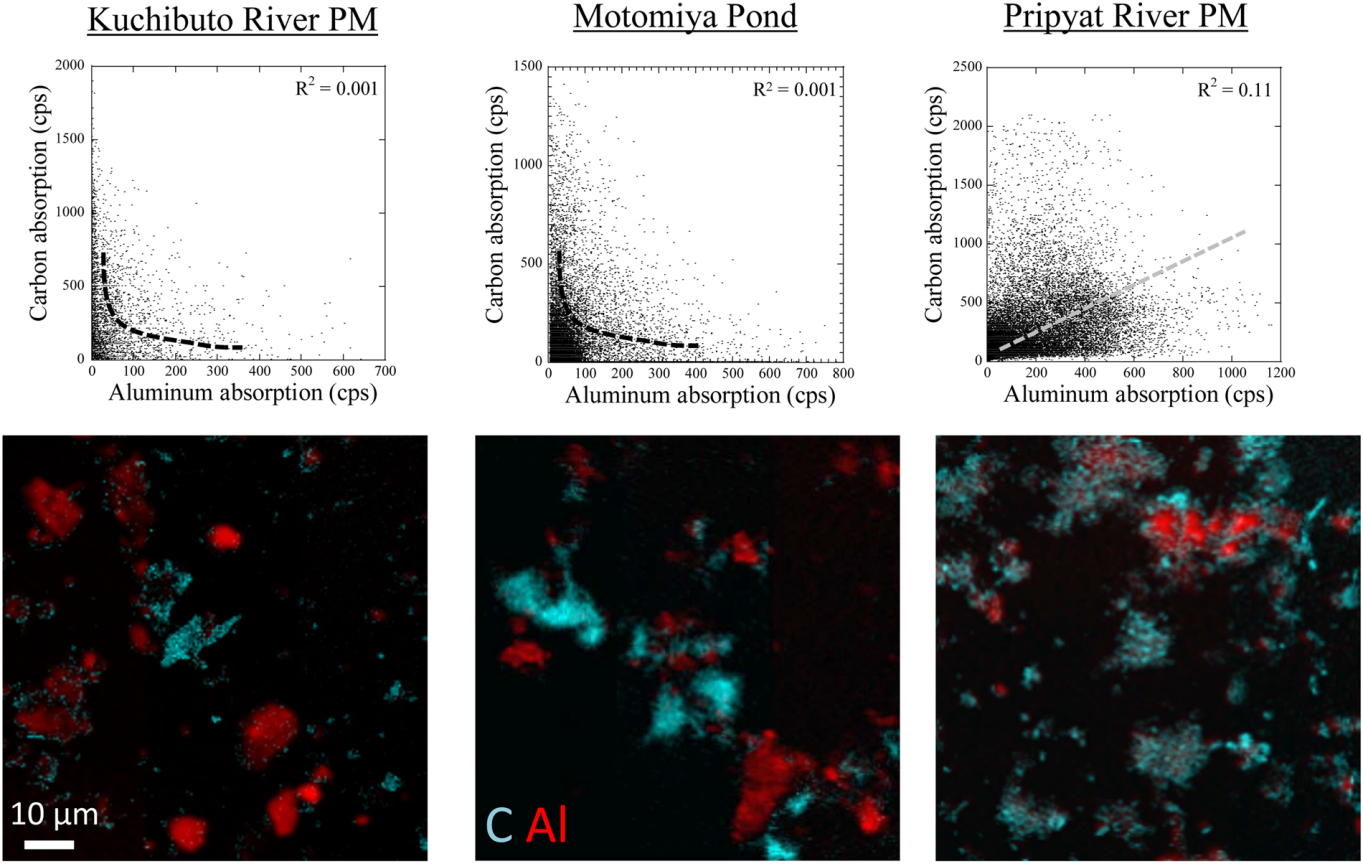
Distributions of Al and C in particulate matters collected in the Kuchibuto and Pripyat Rivers and the Motomiya Pond with their scatter plots.

For better understanding of the association between NOM and particulate matters, STXM analysis for the particulate matters obtained from Pripyat and Kuchibuto Rivers and Motomiya Pond was conducted. For the STXM analysis, particulate matter samples were completely dispersed on a Si_3_N_4_ membrane after thorough sonication treatment of the particulate matters in pure water^35^. Mappings of C and Al were obtained through STXM based on subtraction of optical density images taken at post- and pre-edges for C and Al K-edges, respectively (Fig. 5). In addition, potassium (K) shows a distribution pattern similar to that of Al in the particles (Fig. S2 in Supporting Information), suggesting that Al mostly exists as phyllosilicates in particulate matters. NOM are associated with Al-bearing particles collected from Pripyat River, demonstrating the strong affinity between NOM and clay minerals in Pripyat River, and such strong affinity in turn reduces Cs adsorption. By contrast, STXM images of the particulate matters collected from Kuchibuto River and Motomiya Pond showed that C and Al are more or less independently distributed as particulate matters. The difference between the two rivers is more clearly demonstrated in scattered plots of the absorption intensities of C and Al in their mapping data (Fig. 5): relatively high correlation was found in particulate matters of Pripyat River but not in Kuchibuto River and Motomiya Pond.

**Figure 6:**
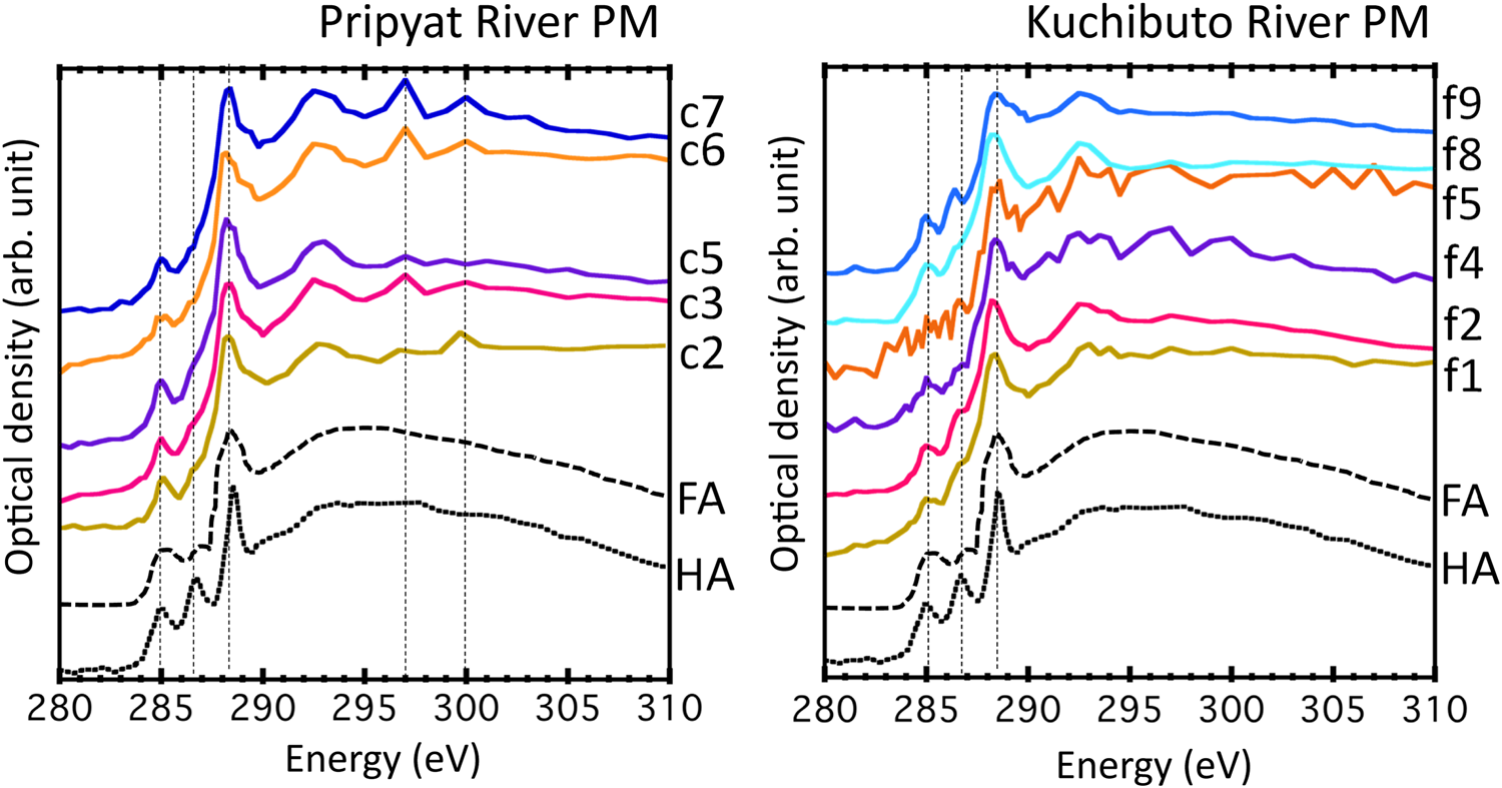
Carbon K-edge NEXAFS spectra at various carbon-rich spots in STXM images for natural organic matter in particulate matters recovered from Pripyat and Kuchibuto Rivers. Spectra of fulvic (FA; Suwannee River fulvic acid^47^) and humic acids (HA; Suwannee River humic acid^47^) were also shown as reference materials. Absorption peaks of 285.1, 286.8, and 288.6 eV correspond to aromatic, phenolic, and carboxylic carbons, respectively. Absorption of potassium L-edges were also found at 297.1 and 299.9 eV for the samples from Pripyat River.

In investigating the speciation of C in the particulate matters, near-edge X-ray absorption fine structure (NEXAFS) spectra at C K-edge were obtained through STXM by using the image stacking method (Fig. 6)^36^. NEXAFS spectra at 5–6 spots are basically similar to those of fulvic and humic acids (HAs), wherein C displays characteristic peaks at 285.1 eV (aromatic C), 286.8 eV (phenolic or ketonic C), and 288.6 eV (carboxylic C). Moreover, spectra of the particulate matters are more similar to those of fulvic acid with lower contribution at 286.8 eV peak. This result is reasonable considering that the particulate matters were collected from freshwater, which contains more soluble fraction of humic substances, or fulvic acid. Hence, NEXAFS confirmed that the dominant species of NOMs covering the particulate matters are humic substances. In addition, absorption of potassium L-edge was prominent only in sample collected from Pripyat River (Fig. 6), further demonstrating the close association between NOM and clay minerals in Pripyat River. Such submicron-scale characterization of NOM is indispensable in revealing the nature of particulate matters in rivers.

Although C K-edge NEXAFS showed that NOM detected in the particulate matters in Kuchibuto and Pripyat Rivers are humic substances, association of humic substances and clay mineral particles was considerably weaker in Kuchibuto River than in Pripyat River. Coagulation of humic substances and clay minerals are strongly enhanced in the presence of divalent cations, such as Ca^2+^ and Mg^2+^, in natural waters^37–39^. Based on the data on water quality (Table 1), Ca^2+^ concentration was higher in Pripyat River (approximately 1.6 mM) than in freshwater samples obtained in Fukushima by a factor of 4.6. This difference was also found as general trends in major ion concentrations in Japanese and Black Sea rivers^40^. In particular, the critical concentration of Ca^2+^ that induces aggregation of humic substances was 1 mM^38,39^. These results can explain the strong association of humic substances and clay minerals in Pripyat River.

### EXAFS for Cs adsorbed on particulate matters after treatment with H_2_O_2_

To confirm the blocking effect of NOM on the particle surfaces to Cs adsorption, changes in Cs adsorption species before and after H_2_O_2_ treatment to remove NOM were evaluated based on EXAFS (Fig. 3). Before the treatment, the CN_IS_/CN_OS_ ratio (=relative importance of IS complex to OS complex in the RSF; Table 3) was considerably larger in Kuchibuto River than in Pripyat River. Even after H_2_O_2_ treatment, the CN_IS_/CN_OS_ ratio for particulate matters in Kuchibuto River was 0.83, slightly higher than that before H_2_O_2_ treatment (0.78; Table 3), suggesting that the effect of NOM is not very important in Cs adsorption on particulate matters in Kuchibuto River. However, the ratio increased dramatically from 0.42 to 1.2 after H_2_O_2_ treatment of the particulate matters (PSP-1) obtained in Pripyat River. These results revealed that (i) Cs is more strongly fixed onto the particulate matters in Kuchibuto River due to the formation of IS complex of Cs with phyllosilicate minerals; (ii) Cs adsorption on particulate matters obtained from Pripyat River is weak due to the inhibition effect of NOM; (iii) phyllosilicate minerals are actually included in the particulate matters in Pripyat River; and (iv) incorporation of Cs into the interlayer can be inhibited by the presence of humic substances. In addition, the XRD patterns displayed the characteristic peaks of phyllosilicate minerals at 6.7° (or 8.5°) and at 12.2° after NOM removal in samples obtained both in Fukushima and Chernobyl (Fig. 1), consistent with the occurrence of high adsorption affinity for Cs after removal of NOM in EXAFS analyses.

### Other factors, including complexation with humic substances, and other types of organic matter

Another possible effect of NOM on solid-water partition is the retention of Cs^+^ in aqueous phase through complexation with humic substances in aqueous phase. Thus, a dialysis method using diffusion cell and HA extracted from paddy soil in Tochigi Prefecture (adjacent to Fukushima Prefecture) was conducted to test the hypothesis^41,42^. This method is detailed in the experimental section, wherein Cs introduced as ^137^Cs gradually diffuses from tracer cell to HA cell (HA concentration: 60 mg/L) as a function of elapsed time (Figs S3 and S4). After equilibrium was established, radioactivity in HA cell finally became higher than that in tracer cell. Blank experiment (n = 2), that is, without HA, reached equal ^137^Cs concentrations between tracer and HA cells. The difference between the two experiments in the absence and presence of HA allows us to determine the stability constant of HA-Cs complex (*β*_MA_), which is defined as^42^:

1 $${\beta }_{{\rm{MA}}}=\frac{[\mathrm{MA}]}{{([{\rm{M}}}^{{\rm{z}}+}][{\rm{A}}])},$$

where z and A are the charges of metal ion and dissociated ligand of HA, respectively. Determination of *β*_MA_ is detailed in Supporting Information. logβ_CsA_ was determined to be 4.4 (pH = 7), which is considerably lower than the logβ_MA_ values determined for divalent cations (e.g., logβ_CaA_ = 7.0 for Ca^2+^) and trivalent cations (12–16 for rare earth ions and Fe^3+^ at pH = 7) for the same HA sample^42^. A logβ_CsA_ value lower than those of multivalent cations is reasonable considering its weak electrostatic attraction between Cs^+^ and HA^42^. The logβ_CsA_ with reported logβ_MA_ values for other cations allows us to conduct speciation calculation of Cs in natural waters in the presence of humic substances including the competitive effect of major multivalent cation (Ca^2+^) on humate formation. The calculation for Cs in Pripyat River was conducted assuming that (i) *DOC* = 50 mg/L, which is higher than that in Pripyat River and (ii) [Ca^2+^] is 64 mg/L following the value in the Pripyat River (Table 1). The estimated Cs-HA fraction in Pripyat River is at most 1% relative to total Cs in water, because complexation of Cs^+^ with HA is strongly inhibited by the presence of Ca^2+^ due to its much higher concentration than Cs^+^ in natural waters. Thus, complexation with HA cannot explain the higher concentration of dissolved Cs^+^ in river water with high *DOC*, such as in Pripyat River.

Naulier *et al*.^43^ have recently suggested that particulate NOM is an important carrier of RCs in Fukushima Rivers. They suggested that NOM consists mainly of initially contaminated dead leaves and/or other allochtonous organic materials, which are abundant in autumn, or during the sampling season in this study. In our study, NOM in particulate matters was characterized as humic substances by STXM, indicating that the NOM examined in this study is not plant debris.

## Discussion

Solid-water partition of RCs dispersed by FDNPP (and CNPP) in river water is important in estimating the subsequent behavior of RCs in environment. For example, large partition into the aqueous phase leads to (i) the incorporation of RCs into the biota or into the ecosystem^4^ and to (ii) the transfer of RCs into ocean through the estuarine area. Moreover, large partition into solid phase results in retention of ^137^Cs in sediments via sedimentation in basins and estuaries^1^. In this study, *K*_*d*_ in Abukuma River System in Fukushima and Pripyat River in Chernobyl were compared, and the result revealed that the *K*_*d*_ values of particulate matters in Abukuma River System is larger than those in Pripyat River by a factor greater than 10‒10^2^, consistent with the findings of Konoplev *et al*.^44^ Moreover, Konoplev *et al*.^44^ gave two possible reasons about the larger *K*_*d*_ in Fukushima: (i)higher value of the radiocesium Interception Potential (RIP) and (ii) presence of water insoluble glassy microparticles containing RCs emitted by the FDNPP accident firstly reported by Adachi *et al*.^45^. However, we think that the second factor cannot fully explain the larger (apparent) *K*_*d*_ value by the RCs-bearing microparticles, because (i) more than half of RCs observed in the aerosol filters collected during the accident were water soluble^23^ and (ii) a preliminary study showed that the contribution of the RCs-bearing microparticles to the activity of total particular matters collected on the filters in the Kuchibuto River were less than 50%^46^. Even if the contribution of the RCs-bearing microparticles was 50%, the log*K*_*d*_ value is apparently increased by only 0.30 (=log2), which cannot explain the larger *K*_*d*_ value by a factor of 10–100 in Fukushima compared with those in Chernobyl. Thus, it is suggested that the larger value can be mainly explained by the larger affinity of RCs to the particulate matters in rivers in Fukushima.

In this study, STXM and EXAFS analyses confirmed that NOM is one of the key factors that control Cs partition in river system, because NOM inhibits the adsorption site on particulate matters and possibly forms a weak OS complex on particulate matters. By using the same experimental protocols applied to the two systems, the present study clearly showed the importance of NOM (and mineralogy) in both systems.

There is a consensus wherein the characteristics of river particulate matters and sediments are dependent on soil type, such as mineral composition and organic content, found around a catchment^1,4,19,20^. In the present study, the different patterns of Cs partition were clarified based on the different mineral compositions and varying NOM contents in Kuchibuto (Fukushima) and Pripyat (Chernobyl) Rivers. In particular, a large amount of NOM present in Chernobyl due to the slow degradation rate of organic matter in cold climate^40^ is responsible for the higher partition of RCs into the aqueous phase in Chernobyl than in Fukushima^5,6,9,20^. Another factor that promotes the association of NOM and clay mineral is the higher [Ca^2+^] (hard water) in Pripyat River than in Kuchibuto River. This difference of [Ca^2+^] in river water can be readily understood by the geologic characteristics of the two areas, since geology in the provenances mainly control water quality of the river^40^: the Pripyat River flows through the Pripyat basin with a carbonate platform (from Devonian to Cretaceous)^47^, whereas the Kuchibuto River through the Abukuma granitic terrain^7,8^, which can explain higher [Ca^2+^] in Pripyat River than in Kuchibuto River.

These results revealed that geological and soil property of provenances and water quality affected by geological and climatic factors control NOM concentration and its association with clay minerals, which in turn affects solid-water partition of RCs in river water. Consequently, the mobility and bioavailability of RCs should be considerably higher in Chernobyl than in Fukushima with regard to the high dissolved form of RCs. This phenomenon is possibly related to the lower probability of introduction into the food chain of RCs produced from the FDNPP accident compared with those produced from the CNPP accident.

## Methods

### Samples and chemicals

The east longitude and north latitude of the sampling site and the physicochemical information of each site are summarized in Table 1. Pripyat River, which is the largest stream near CNPP, is approximately 761 km long in the zone and contains radionuclides released from the CNPP accident (Fig. S1)^9,20^. Particulate matter samples (PSP-1 and PSP-2) were collected in Pripyat River in August 2013 by using a filtration system. Sixty liters of river water was filtered initially through a 3.0 μm membrane filter and then through a 0.45 μm membrane filters by using a pressurized pumping system.

The Kuchibuto River is one tributary of the Abukuma River, which is the largest river in Fukushima Prefecture, Japan (Fig. S1). Main RCs source is the Yamakiya District, Kawamata Town, which is located within the evacuation zone (approximately 30 km from the FDNPP; Fig. S1. Pond sediments were sampled from surface to 2 cm in depth.

Details of chemical analyses of major elements, ^133^Cs, and *DOC* for water samples were given in Table 1. The activities of ^134^Cs and ^137^Cs were measured by non-destructive gamma-ray spectrometer (GC4018/7915-30/ULB-GC, CANBERRA), details of which were given in Supporting Information. The ^134^Cs/^137^Cs activity ratios as to March 11, 2011 were around 1 for Fukushima samples, suggesting that radiocesium was originated from the Fukushima accident^6,33^.

A carrier-free tracer of ^137^Cs in 0.1 mol/L HCl solution used for the diffusion cell experiment was purchased from Eckert & Ziegler Company, USA. Vermiculite (Kent, Connecticut) used as a standard material for EXAFS analysis was purchased from the World’s Natural Science Establishment (Rochester, NY, USA). All chemicals used in experiments were analytical grade without any purification or pretreatment. Standard humic and fulvic acids (the Suwannee River humic and fulvic acids, respectively) received from International Humic Substances Society^48^ were used for reference materials in STXM analysis.

### Preparation of Cs-adsorbed samples for EXAFS measurement and analysis

To understand the effect of NOM on RCs species, several NOM-rich sediments and particulate matters were treated with 35% H_2_O_2_ solution^22,25^. Samples before and after NOM removal by H_2_O_2_ were saturated with 1.0 mol/L CsCl solution for EXAFS analysis. After aging for one week, solid phases were separated through filtration, rinsed with Milli-Q water for three times, and sealed in a polyethylene bag for EXAFS measurement.

Cesium L3-edge EXAFS spectra were obtained on BL-12C or BL-9A at KEK Photon Factory (Tsukuba, Japan). EXAFS analysis was conducted using REX 2000 (Rigaku Co.) and FEFF 7.0^49,50^. After extracting EXAFS oscillation from the raw spectra, RSFs were obtained from *k* ^3^ *χ*(*k*) oscillation by Fourier transform within a *k*-range of 2.2 Å^−1^ to 6.4 Å^−1^. The backscattering amplitudes and phase-shift functions of Cs-O and Cs-Si were extracted by FEFF 7.0 based on the structures of CsOH and Cs_2_Si_2_O_5_, respectively^51,52^. Details of the EXAFS analysis were similar to those previously described^7,12^.

### STXM

Submicron distribution images of C, Al, and K coupled with carbon K-edge NEXAFS spectra of the particulate matters collected in Pripyat and Kuchibuto Rivers were obtained in transmission mode by using a compact STXM at BL-13A in Photon Factory^36^. The particulate matter samples (<0.5 mg) dispersed thoroughly in Milli-Q water (10 mg) with sonication was deposited on a Si_3_N_4_ membrane (50 nm thick) and then air-dried^35^. The membrane was placed on a piezo-driven stages in a chamber purged with 0.1 atm He. The sample was scanned against X-ray beam focused by a Fresnel zone plate (beam size: less than 30 mm × 30 nm). Mapping of transmitted X-ray from pre-edge to post-edge of carbon K-edge, potassium L-edge, or aluminum K-edge was recorded using the image stacking method^36^. NEXAFS spectra at each edge were obtained using the aXis2000 software^36^.

## Electronic supplementary material

Supporting Information
