# Supplementary material for: Comparison of Solid-Water Partitions of Radiocesium in River Waters in Fukushima and Chernobyl Areas
Source: Sci Rep. 2017 Sep 29;7:12407. doi: 10.1038/s41598-017-12391-7 (PMC5622054; doi:10.1038/s41598-017-12391-7)
Supplement: Supplementary file 1 — Supporting Information [file 41598_2017_12391_MOESM1_ESM.pdf]

Supporting Information for

Comparison of Solid-Water Partitions of Radiocesium in River Waters in Fukushima and Chernobyl Areas

Yoshio Takahashi,[1,2] Qiaohui Fan,[1,3] Hiroki Suga,[4] Kazuya Tanaka,[5] Aya Sakaguchi,[6] Yasuo Takeichi,[2] Kanta Ono,[2] Kazuhiko Mase,[2] Kenji Kato,[7] and Vladimir V. Kanivets[8]

[1]Department of Earth and Planetary Science, The University of Tokyo, Hongo 7-3-1, Bunkyo-ku, Tokyo, 113- 8654, Japan

[2] Institute of Materials Structure Science, High-Energy Accelerator Research Organization (KEK), Oho, Tsukuba, Ibaraki 305-0801

[3] Northwest Institute of Eco-Environment & Resources, CAS, 382 West Donggang Road, Lanzhou, Gansu, 730000, China

[4] Department of Earth and Planetary Systems Science, Hiroshima University, Kagamiyama, Higashi-Hiroshima, Hiroshima 739-8526

[5]Advanced Science Research Center, Japan Atomic Energy Agency, Tokai, Ibaraki 319-1195, Japan

[6] Center for Research in Isotopes and Environmental Dynamics, University of Tsukuba, 1-1-1 Tennodai, Tsukuba, Ibaraki 305-8577, Japan

[7] Department of Geosciences, Faculty of Science, Shizuoka University, 836 Ohya, Suruga-ku, Shizuoka 422-8529

[8] Ukranian Hydrometeorological Institute, Nauka, 37, Kiev, Ukraine

Chemical composition analyses of water samples.

Concentrations of Na^+, NH_4^+, K^+, Mg^{2+}, Ca^{2+},Cl^-, NO_3^-, and SO_4^{2-} were measured by ion chromatography (ICS-1100, Dionex, Japan), while that of ^{133}Cs was measured by inductively coupled plasma mass spectrometry (ICP-MS, Agilent 7700, Agilent, Japan) using ^{115}In as an internal standard at the concentration of 1 ppb.

Cesium-134 and ^{137}Cs were measured using a planar-type Ge detector (GC4018/7915-30/ULB-GC, CANBERRA) by the gamma-rays of the 604 keV and 662 keV. Details of the method were given in Sakaguchi et al.[5] The spectrometer was calibrated with (1) a standard which was prepared by the Low Level Radioactivity Laboratory of Kanazawa University using NBL (New Brunswick Laboratory) reference material No.42-1 (4.04% U), (2) a γ-ray certified reference material (JSAC 0471) including ^{134}Cs and ^{137}Cs from the ASAC (the Japan Society for Analytical Chemistry) and (3) KCl of analytical grade.

X-ray diffraction (XRD) patterns were measured with a powder X-ray diffractometer (MultiFlex, Rigaku Co.) using CuKα radiation at 40 mA and 40 kV.

Inorganic and organic carbon contents in the water samples were measured by total carbon analyzer (Shimadzu TOC-VWS, Kyoto, Japan), while total organic carbon in the solid samples was measured by elemental analyzer (Vario MICRO; Elementar, Germany).

Diffusion cell method for the determination of stability constant of Cs-humate species.

Stability of Cs-humate complex was evaluated by equilibrium dialysis using diffusion cell method.[38] In this study, competition ligand employed in Van Loon et al.[41] was not used, since complexation of Cs^+ is weak, by which Cs can be distributed in the both cells even in the presence of humic acid (HA). A HA sample (THA) extracted from paddy field in Tochigi Prefecture, Japan was used in this experiment. Details and characterization of the HA sample were given elsewhere (Takahashi et al., 1995). The complexation reaction of HA with metal ion (M^{z+}) can be written as

$$M^{z+} + A \leftrightarrow MA \tag{1}$$

and the stability constant, β_{MA}, can be expressed as

$$\beta_{MA} = \frac{[MA]}{([M^{z+}][A])}, \tag{2}$$

where z and A are the charge of metal ion and dissociated ligand of HA, respectively.[42] In general, the β_{MA} has to consider multidentate sites other than monodentate sites. As the binding sites in the polyelectrolyte are fixed on the framework, we assumed that a multidentate site consisting of neighboring functional groups behaves as a unit of functional group in HA. Therefore, [A] in Eq. (2) is combination of the concentrations of both monodentate and multidentate sites. The apparatus consists of tracer (trc) and HA cells (Fig. S3); ^{137}Cs tracer was added to the former cell, while HA was introduced into the latter cell. Based on the inspection of UV-vis absorption, it was confirmed that HA did not flow through the membrane filter due to the large molecular weight. Thus, we can write ^{137}Cs concentrations in the two cells as

$$[^{137}Cs]_{trc} = [^{137}Cs]_{free}$$
$$[^{137}Cs]_{HA} = [^{137}Cs]_{free} + [^{137}Cs\text{-}A].$$

Consequently, the stability constant (β_{CsA}) can be written as:

$$\beta_{CsA} = [^{137}CsA] / ([^{137}Cs]_{free}\,[A])$$
$$= ([^{137}Cs]_{HA} - [^{137}Cs]_{trc}) / ([^{137}Cs]_{trc}\,[A]).$$

Since (i) [A] at various pH was determined by pH titration and (ii) consumption of [A] by Cs is negligible, [A] can be determined in the HA cell, which leads to the determination of β_{CsA}. Equilibrium of the method was attained within about three weeks. It was also confirmed that ^{137}Cs was equally distributed to the both cells when ^{137}Cs was initially added to one of the cells in the absence of HA (Fig. S4).

References

MEXT (2011) http://radioactivity.nsr.go.jp/ja/contents/5000/4901/24/1910_1216.pdf

Erig Gaba, Map of the radiation hotspots of Cesium-137 in 1996 resulting from the Chernobyl nuclear power plant accident. *In Wikipedia: The Free Encyclopedia*. Retrieved from https://commons.wikimedia.org/wiki/File:Tchernobyl_radiation_1996.svg (2006).

Takahashi, Y., Minai, Y., Kimura, T., Meguro, Y. & Tominaga, T. Formation of actinide (III)-humate and its influence on adsorption on kaolinite. *Mat. Res. Soc. Symp. Proc.* **353**, 189-196 (1995).

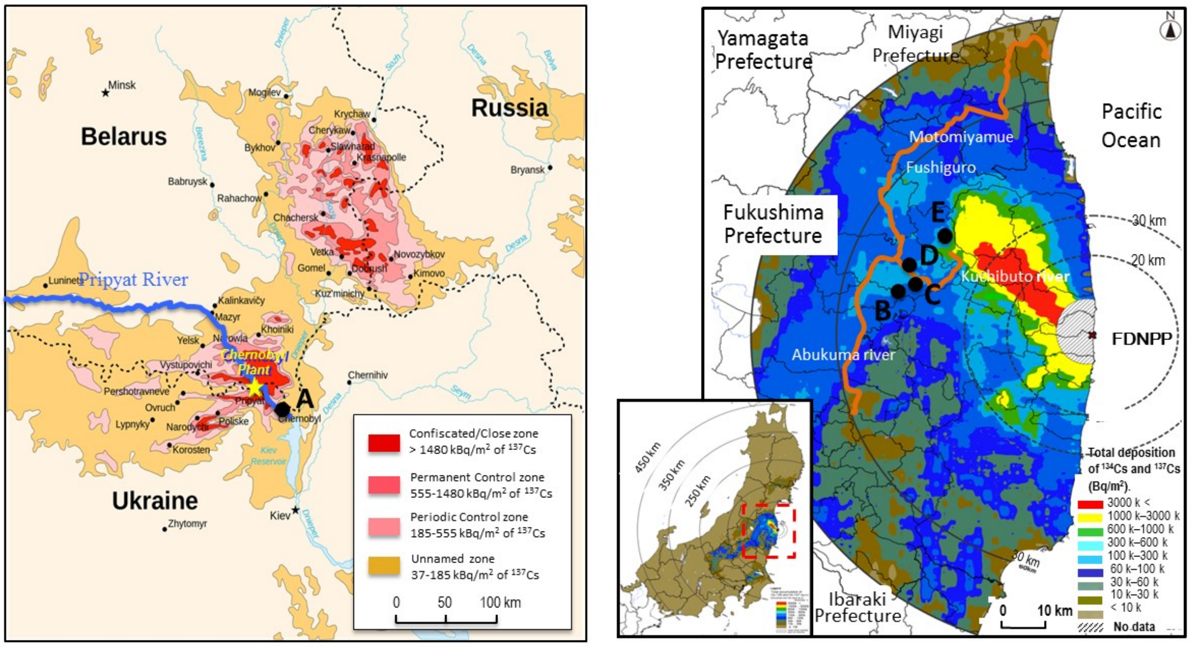

Figure S1. Sampling sites in Chernobyl (left figure; modified from Eric Gaba, 2006; Source: https://commons.wikimedia.org/wiki/File:Tchernobyl_radiation_1996.svg created by Eric Gaba. This file is licensed under the Creative Commons Attribution-Share Alike 2.5 Generic license, https://creativecommons.org/licenses/by-sa/2.5/deed.en; Some changes (indication of "A" and change the radioactivity unit from Ci to Bq) were made to the original file) and Fukushima (right figure; modified from MEXT, 2011; Source: Nuclear Regulation Authority website (http://radioactivity.nsr.go.jp/ja/contents/5000/4901/24/1910_1216.pdf)). (A) The Pripyat River around CNPP; (B) Motamiya Pond; (C) Ooyada Pond; (D) Downstream of the Kuchibuto Rive; (E) Matsuzawakami Pond.

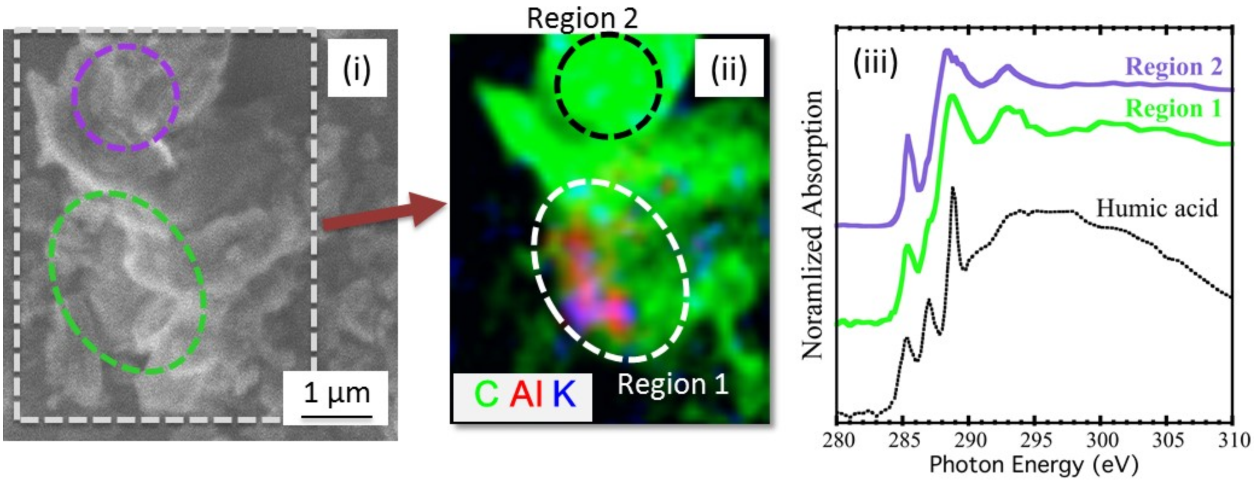

Figure S2. Distributions of carbon (C), aluminum (Al), and potassium (K) and C K-edge NEXAFS spectra of organic C in the map. (i) Back-scattered electron image of particulate matters collected in Pripyat River; (ii) STXM mapping of C, Al, and K; (iii) C K-edge NEXAFS spectra of Regions 1 and 2 shown in (i) and (ii).

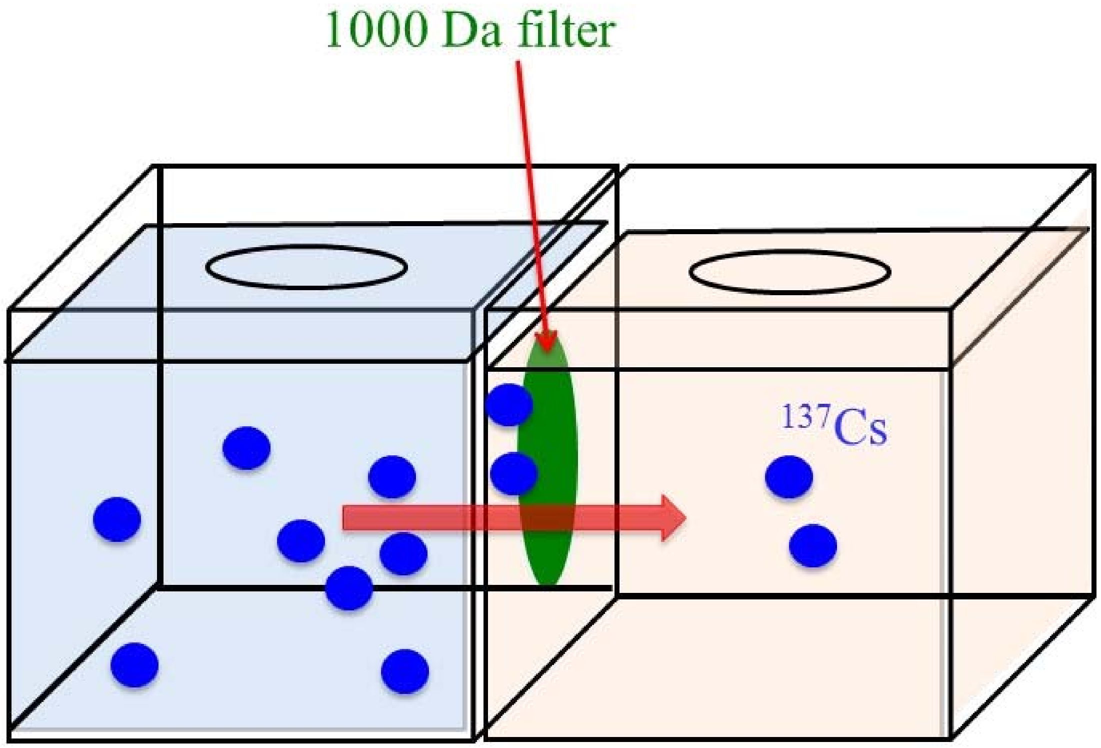

Figure S3. The diffusion equipment for ^{137}Cs diffusion experiment in carrier-free system. pH=7.0, I=0.010 mol/L NaCl solution.

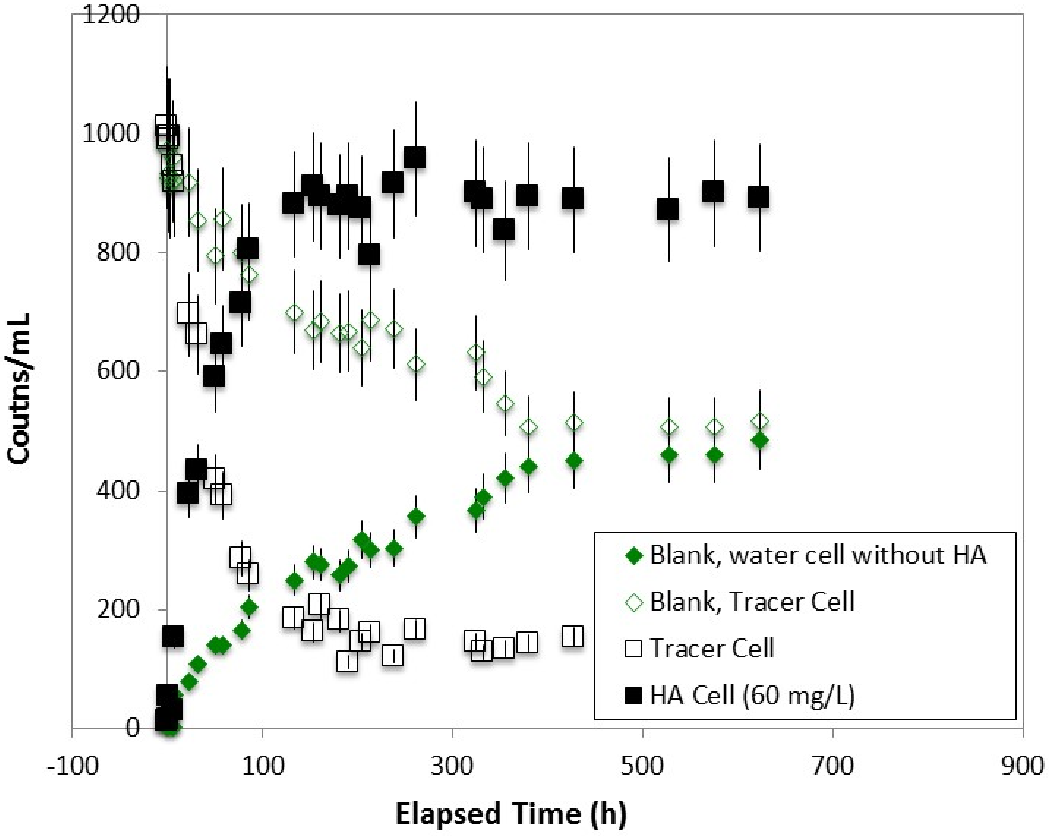

Figure S4. Diffusion of ^{137}Cs from tracer cell to HA cell as a function of elapsed time (pH=7.0; T=25 ^{o}C; concentration of HA: 60 mg/L).
